# Supplementary material for: Fucose-Rich Sulfated Polysaccharides from Two Vietnamese Sea Cucumbers Bohadschia argus and Holothuria (Theelothuria) spinifera: Structures and Anticoagulant Activity
Source: Mar Drugs. 2022 Jun 6;20(6):380. doi: 10.3390/md20060380 (PMC9228488; doi:10.3390/md20060380)
Supplement: Supplementary file 1 [file marinedrugs-20-00380-s001.zip › marinedrugs-1743179-supplementary.pdf]

Supplementary data

Fucose-rich sulfated polysaccharides from two Vietnamese sea cucumbers *Bohadschia argus* and *Holothuria (Theelothuria) spinifera*: structures and anticoagulant activity

Nadezhda E. Ustyuzhanina, Maria I. Bilan, Andrey S. Dmitrenok, Eugenia A. Tsvetkova, Sofya P. Nikogosova, Cao Thi Thuy Hang, Pham Duc Thinh, Dinh Thanh Trung, Tran Thi Thanh Van, Alexander S. Shashkov, Anatolii I. Usov and Nikolay E. Nifantiev

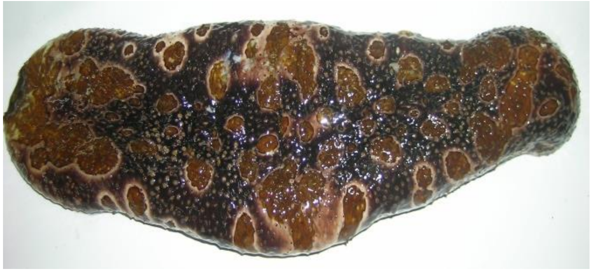

A

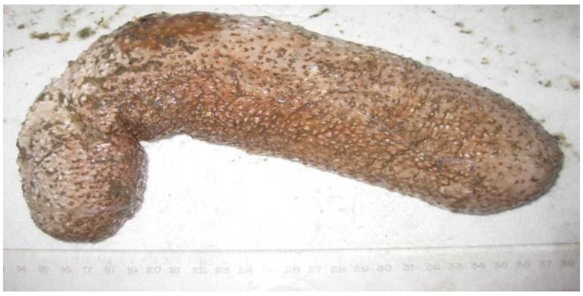

B

Figure S1. Sea cucumbers (**A)** *Bohadschia argus* (Jaeger, 1833) and (**B)** *Holothuria (Theelothuria) spinifera* (Theel, 1886)

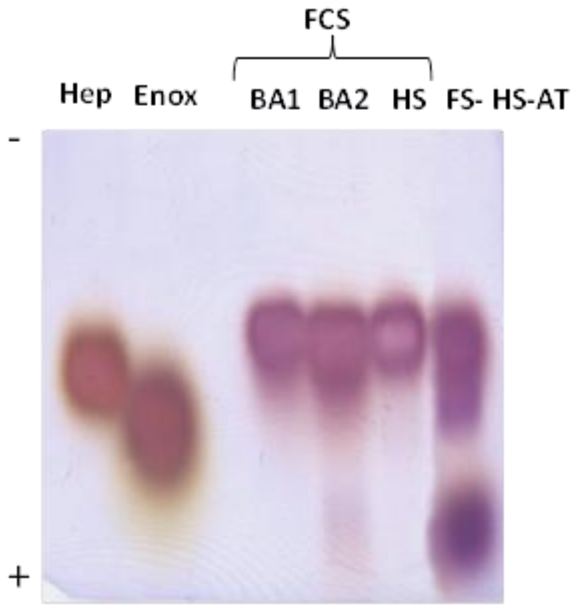

Figure S2. Agarose gel electrophoresis of polysaccharides. Abbreviations: **Hep** – unfractionated heparin from Sigma; **Enox** – enoxaparin (Clexane®, Sanofi); **BA1, BA2** – *Bohadschia argus* **FCS** fractions eluted from DEAE-Sephacel with 0.75 M and 1.0 M NaCl, respectively; **HS** – the corresponding *Holothuria spinifera* **FCS** fraction eluted from DEAE-Sephacel with 1 M NaCl; **FS-HS-AT** – fraction of **FS** obtained from crude polysaccharide of *Holothuria spinifera* after mild acid treatment and ion-exchange chromatography.

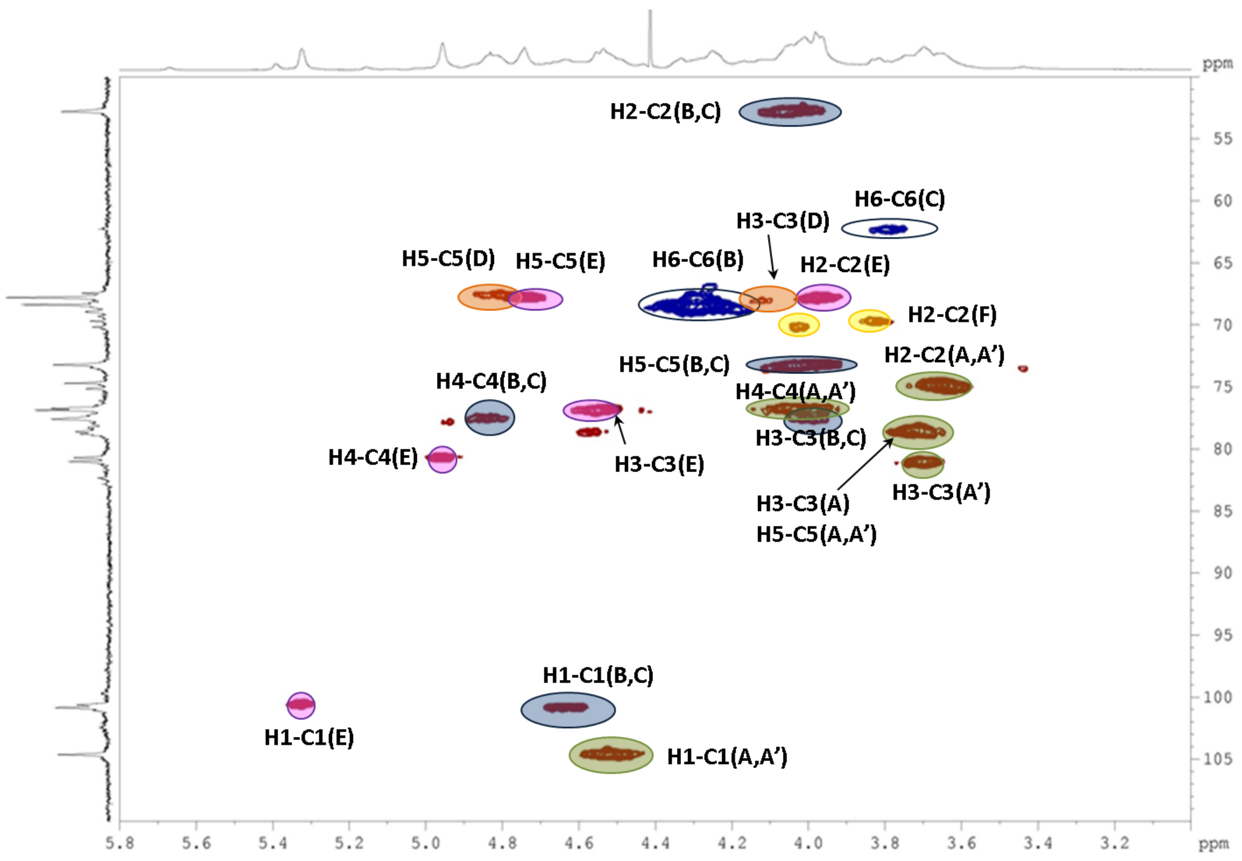

Figure S3. The HSQC NMR spectrum of **FCS-BA**

Table S1. The data of the ^{1}H and ^{13}C NMR spectra (chemical shifts, ppm) of fucosylated chondroitin sulfates

Residue	H-1<br>(C-1)	H-2<br>(C-2)	H-3<br>(C-3)	H-4<br>(C-4)	H-5<br>(C-5)	H-6<br>(C-6)
**A**→4)-β-D-Glc*p*A-(1→	4.48	3.64	3.71	3.96	3.71	-
	(105.0)	(75.0)	(78.2)	(76.6)	(78.2)	(176.0)
**A'**→4)-β-D-Glc*p*A-(1→	4.48	3.60	3.68	4.00	3.71	-
	(105.0)	(75.0)	(80.7)	(76.6)	(78.2)	(176.0)
**B**→3)-β-D-Gal*p*NAc4*S*6*S*-(1→	4.58	4.07	3.95	**4.81**	4.00	**4.33, 4.20**
	(100.9)	(52.7)	(77.9)	**(77.2)**	(73.2)	**(68.5)**
**C**→3)-β-D-Gal*p*NAc4*S*-(1→	4.58	4.07	3.95	**4.81**	4.02	3.81
	(100.9)	(52.7)	(77.9)	**(77.2)**	(76.2)	(62.3)
**D** α-L-Fuc*p*2*S*4*S*-(1→	5.69	**4.48**	4.17	**4.86**	4.90	1.37
	(97.7)	**(76.6)**	(67.8)	**(82.5)**	(67.5)	(16.9)
**E** α-L-Fuc*p*3*S*4*S*-(1→	5.34	3.95	**4.53**	**5.01**	4.85	1.37
	(100.5)	(67.6)	**(76.6)**	**(80.6)**	(67.6)	(17.2)
**F** α-L-Fuc*p*4*S*-(1→	5.41	3.82	4.04	**4.77**	4.85	1.37
	(99.6)	(69.7)	(70.0)	**(82.4)**	(67.6)	(17.2)

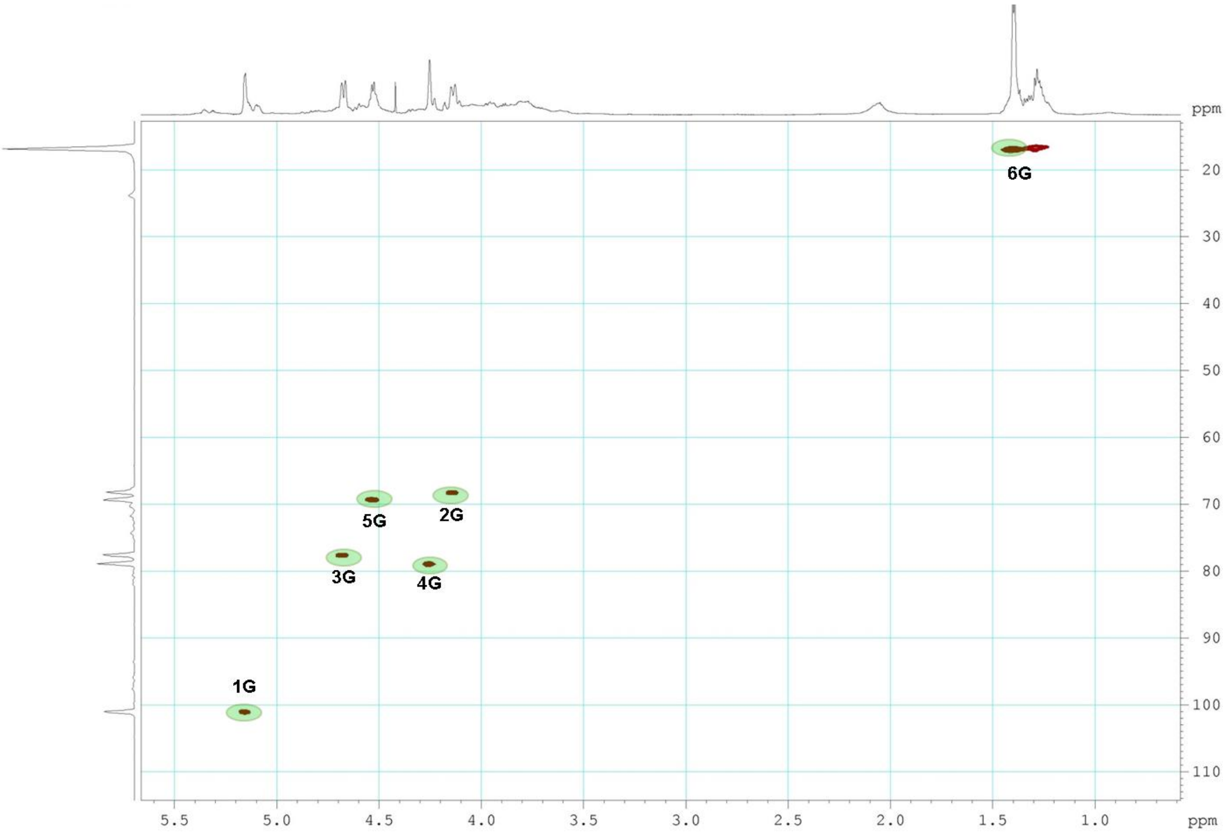

Figure S4. The HSQC NMR spectrum of **FS-BA-AT**

Table S2. The data of ^{1}H and ^{13}C NMR spectra (chemical shifts, ppm) of sulfated fucan **FS-BA-AT** and its desulfated derivative **FS-BA-AT-DS**

Species, sample	Structural unit	H1/C1	H2/C2	H3/C3	H4/C4	H5/C5	H6/C6
Holothuria fuscopunctata [22]	-4)-α-L-Fuc*p*3S-(1-	5.22<br>103.00	4.20<br>69.88	4.73<br>79.28	4.21<br>80.71	4.62<br>71.17	1.47<br>18.68
**FS-BA-AT**	**G** -4)-α-L-Fuc*p*3S-(1-	5.15<br>101.00	4.14<br>68.3	4.67<br>77.6	4.26<br>78.9	4.53<br>69.3	1.40<br>16.9
	α-L-Fuc*p*3S-(1-	5.10<br>101.1	3.97<br>67.9	4.61<br>79.0	4.18<br>71.7	4.57<br>68.0	1.28<br>16.5
**FS-BA-AT-DS**	-4)-α-L-Fuc*p*-(1-	5.11<br>96.83	4.03<br>69.71	3.96<br>67.58	4.00<br>76.22	4.31<br>67.79	1.22<br>16.29

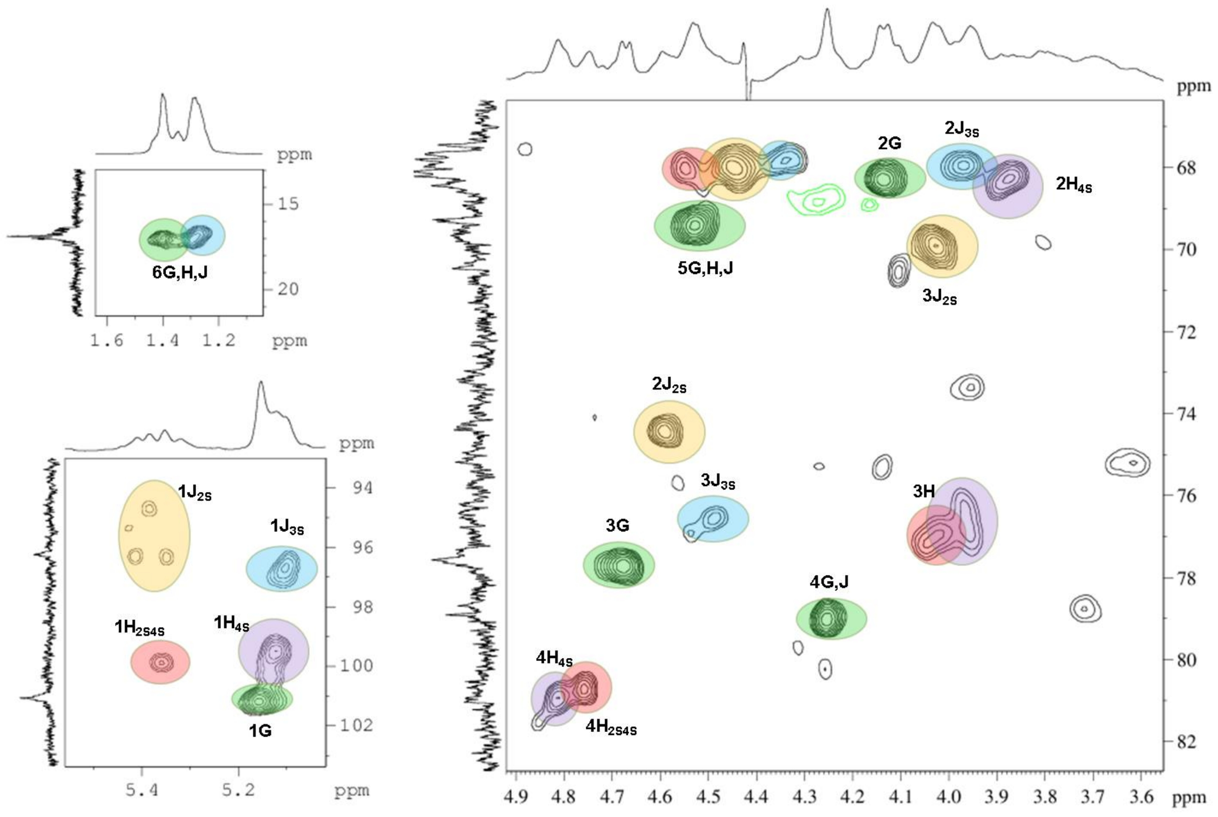

Figure S5. The HSQC NMR spectrum of **FS-HS-AT**

Table S3. ^{13}C and ^{1}H chemical shifts δ, ppm of anomeric atoms in the NMR spectra of fucan sulfate **FS-HS-AT** (see Figure 6)

Residue	$\mathbf{J}_{2S}\rightarrow H_{2,4S}$	$H_{2,4S}\rightarrow\mathbf{J}_{2S}\rightarrow H_{4S}$	$H_{4S}\rightarrow\mathbf{J}_{2S}\rightarrow H_{4S}$	$\mathbf{J}_{3S}\rightarrow H$	$\mathbf{H}_{2,4S}\rightarrow J$	$\mathbf{H}_{4S}\rightarrow J_{3S}$	$\mathbf{H}_{4S}\rightarrow J_{2S}$
C-1	94.7	96.3	96.3	96.7	99.9	99.5	100.4
H-1	*5.39*	*5.41*	*5.32*	*5.10*	*5.36*	*5.12*	*5.14*
